# Th-1 cytokine-primed small extracellular vesicles from limbal mesenchymal stem cells modulate immune and inflammatory responses in Hashimoto's Thyroiditis: an ex vivo proof-of-concept study

**DOI:** 10.1186/s43556-025-00388-y

**Published:** 2025-12-15

**Authors:** Laura Tomasello, Valentina Guarnotta, Giuseppe Siragusa, Mattia Biondo, Giorgio Arnaldi, Carla Giordano, Giuseppe Pizzolanti

**Affiliations:** 1 Department of Health Promotion, Mother and Childcare, Internal Medicine and Medical Specialties, Laboratory of Endocrinology and Regenerative Medicine “Aldo Galluzzo”, University of Palermo, Piazza Delle Cliniche 2, Palermo, 90127 Italy; 2 University Hospital “Paolo Giaccone”, Piazza Delle Cliniche 2, Palermo, 90127 Italy; 3 Scienze E Tecnologie Biologiche Chimiche E Farmaceutiche (STEBICEF), University of Palermo, Viale Delle Scienze, Ed. 16, Palermo, 90128 Italy; 4 ATeN Centre-Advanced Technologies Network Centre, Viale delle Scienze, Ed. 18, Palermo, 90128 Italy

Dear Editor,

Hashimoto’s thyroiditis (HT) is the most prevalent autoimmune thyroid disorder characterized by extensive lymphocytic infiltration and impaired regulatory T cell (Treg) function. Therefore, some add-on therapy including Vitamin D, Selenium and JAK-inhibitor aimed at modulating the immunoinflammatory response, are being explored. Additionally, restoring immunotolerance represents a promising therapeutic strategy [1]. Recently, both in vitro and in vivo preclinical models, along with emerging clinical studies have highlighted the potential of small extracellular vesicles (sEVs), membrane-bound particles ≤ 200 nm secreted by cells into the extracellular space, for the treatment of inflammatory diseases due to their ability to influence a wide spectrum of biological processes in target cells. However, the lack of standardized protocols for sEV isolation and storage remains a critical limitation, as these factors can substantially affect vesicle features, including cargo content and bioactivity. Furthermore, the extent to which sEVs can faithfully convey the immunomodulatory properties of their parent mesenchymal stromal cells (MSCs) is still under investigation [2].

In our previous work, we shown that Th1-primed fibroblast limbal mesenchymal stem cells (f-LSCs) exert stronger immunosuppressive effects than bone marrow-derived MSCs in HT patients, by modulating key mediators such as indoleamine 2,3 dioxygenase (IDO) and programmed death ligand (PD-L)−1 [2]. f-LSCs represent a unique MSC source, derived from an immune-privileged site, the limbus (online resource). Additionally, we suggest that MSCs from this source exhibit enhanced immunomodulatory functions compared to bone marrow derived MSC, and we also proposed a molecular mechanism involving heterogeneous nuclear ribonucleoprotein (hnRNP)-A2/B1 [3].

To explore whether the immunomodulatory effects of f-LSCs could be recapitulated by their derived sEVs, we evaluated the ability of their sEVs to modulate immune responses in activated peripheral blood mononuclear cells (PBMCs) of patients suffering from HT; moreover, we assessed whether f-LSC-derived sEVs could transfer this immunoregulatory potential. Detailed information on participant recruitment and laboratory procedures is available in supplementary material.

sEVs were isolated using two different methods, including tangential flow filtration (TFF) and chemical precipitation (CP). Isolated vesicles were subsequently lyophilized via a one-step freeze-drying protocol. We performed a comprehensive characterization of vesicle yield, size, and purity using dynamic light scattering (DLS), protein quantification, western blotting, and flow cytometry for canonical EV markers. Following lyophilization, both TTF (TFFlyo) and by CP (CPlyo) derived nanoparticles shown detectable fluorescence after CFSE staining and exhibited DLS profiles comparable to freshly isolated nanoparticles, with average particle size of 136.31 ± 15.69 nm and 94.13 ± 6.96 nm vs. 133.52 ± 8.71 nm and 133.02 ± 11.68 nm (*p* > 0.05, Fig. 1a). The polydispersity index (PDI), however, appeared markedly affected by the isolation method: TFFlyo maintained a more regular size reporting a PDI within a 0.38–0.45 range, whereas CPlyo displayed a broader heterogeneity (PDI within 0.97–1 range). Protein quantification suggested that CP yielded a higher amount of particle compared to TFF method (data not shown), whereas western blotting confirmed the sEVs identity of the isolated nanoparticles and revealed that TFF isolation method enriched in CD81, CD63 and CD9, while maintaining unchanged Alix protein levels (online resource). Interestingly, the lyophilization did not alter the expression of key sEV markers CD63 and CD81 (667.4 ± 15.17 µg/mL vs. 345.8 ± 50.87 µg/mL, p0.05, 89 vs. 17.98 ± 2.1 and 68.07 ± 9.45, p > 0.05, online resource), when compared to both CP and TFF- isolated fresh sEVs.

**Fig. 1:**
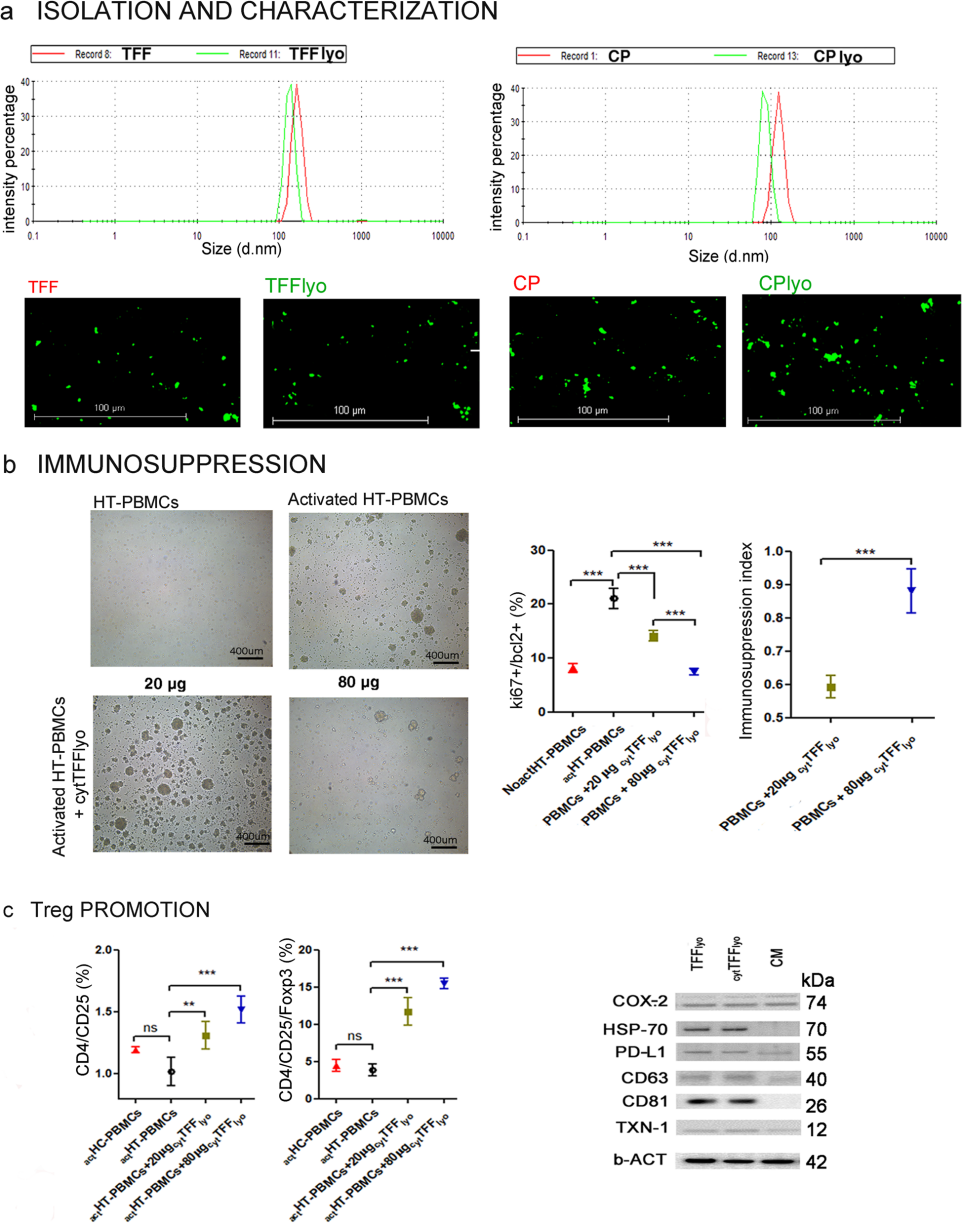
In vitro immunomodulatory effect of Th-1 primed sEVs on activated PBMC from HT suffering patients. **a** comparative analysis of Dynamic Light Scatterer distribution by the intensity determination (percentage, %) and size measurement (nanometer of diameter, d.nm) and immunofluorescence microscopy of sEV derived from f-LSCs isolated by tangential flow filtration (TFF, left panel) and chemical precipitation (CP, right panel), (Carboxyfluorescein succinimidyl ester-CFSEs in green fluorescence, 20x, scale bar 100 µm). The histogram graphs are representative of five independent isolation experiments (biological replicates); **b** Representative images of PBMC from healthy donors and patient suffering from HT under optical microscopy observation after activation and treatment with different amount of EVs (left panel); mean and standard deviation graph representation of cytometric evaluation of the percentage ki67/bcl2 double positive cell population in no activated PBMCs, anti CD3/CD28 activated PBMCs; anti CD3/CD28 activated PBMCs treated with 20 μg cytTFFlyo; anti CD3/CD28 activated PBMCs treated with 80 μg cytTFFlyo (ki67/bcl2% indicates the cell population in proliferation, specifically ki67 positive fraction represent the percentage of cells actively dividing and bcl2 positive fraction indicates the percentage of cells resistant to apoptosis); mean graph representation of the measure of immunosuppressive proliferation index (ISI) in no activated PBMCs, anti CD3/CD28 activated PBMCs; anti CD3/CD28 activated PBMCs treated with 20 μg cytTFFlyo; anti CD3/CD28 activated PBMCs treated with 80 μg cytTFFlyo (right panel); **c** the density graphs represent flowcytometric analysis of activated PBMCs collected by healthy controls (HC) and Hashimoto’s Thyroiditis (HT) patients with or without 20 μg or 80 μg cytTFFlyo stained for CD24, CD25 and Foxp3, data are represented as the percentage of CD4/CD25 double positive and CD4/CD25/Foxp3 triple positive population (left panel). Western blot analysis: the gel bands of HSP-70, PD-L1, COX-2, TXN-1, CD63, CD81 and β-Actin, as normalized protein in sEVs lyophilized Th1-primed and no treated (respectively in first and second line of gel) and in culture medium (CM, in third line of gel) (right panel).The histogram and density graphs were performed by GraphPad Software, Inc, California and are represented as mean value and ± SD; *p* = *p* value, * ≤ 0.05, ** ≤ 0.01, *** ≤ 0.001, ns = no significant (*p* > 0.05). TFF: tangential flow filtration; CP: Chemical precipitation; cyt: cytokines; lyo: lyophilized, TFF: tangential flow filtration; CP: Chemical precipitation. cyt: cytokines; lyo: lyophilized

Given the practical advantages of TFF, reducing both instrumentation complexity and operational costs, TFF was selected for functional assays. We assessed the effects of TFF-isolated and Th-1 primed sEVs on activated PBMCs derived from patients suffering from HT (HT-PBMCs;). Analysis of the cell cycle distribution (online resource), proliferating/surviving T cells population (as percentage of Ki-67/Bcl-2 double-positive cell fraction) and immunosuppressive index (as ISI: $\frac{\%\mathrm{PBMCs}\;\mathrm{Activated}-\%\;\mathrm{PBMCs}\;\mathrm{Activated}\;\mathrm{and}\;\mathrm{treated}}{\left(\%\mathrm{PBMCs}\;\mathrm{Activated}-\%\;\mathrm{PBMCs}\;\mathrm{No}\;\mathrm{treated}\right)}$) demonstrated that Th1-cytokines primed EVs significantly suppressed T-cell expansion in a dose dependent manner. In detail, the percentage of cells in G2/S phase were 14.28 ± 1.71% (ISI 0.59 ± 0.03, p < 0.001) and 9.03 ± 1.04% (ISI 0.88 ± 0.06, p < 0.00) respectively in _act_HT-PBMCs treated with 20 µg and 80 µg _cyt_TFF_lyo_ in comparison to 27.39 ± 1.63% in untreated _act_HT-PBMCs (Fig. 1b). Moreover, a dose dependent increase in the percentage of the main phenotype of active Treg cells, recognized as CD4^+^/CD25^+^/Foxp3^+^ cells within the CD4^+^/CD25^+^ gated population, was observed after treatment with _cyt_TFF_lyo_- sEVs. Specifically, the Treg frequency was 11.71 ± 1.85% and 15.44 ± 0.71% in 20 µg, 80 µg _cyt_TFF_lyo_ respectively, compared to 3.88 ± 0.80% in untreated _act_HT-PBMCs (p < 0.001; Fig. 1c). Consistently, gene expression analysis in 20 µg, 80 µg _cyt_TFF_lyo_ treated HT-PBMCs shown upregulation of IDO1, PD-L1 and interleukin (IL)−4 mRNA, along with a concomitant downregulation of pro-inflammatory mediators, including IL-2, IL-17, monocyte chemoattractive protein (MCP)−1, and hnRNP-A2/B1, a factor previously implicated in immunotolerance induction compared to untreated _act_HT-PBMCs (online resource) (1). Notably, EV cargo analysis of sEVs revealed that Th1 priming enhanced the vesicular content of several molecules. An increased expression of several immunoregulatory proteins was observed in _cyt_TFF_lyo_ -sEVs, but not in TTF-sEVs, compared to their levels in the culture medium (CM). Specifically, COX-2, a known inducer of Foxp3 and Treg promotion, was upregulated by approximately 1.4-fold; thioredoxin-1 (TXN-1), a redox regulator that may support Treg function under inflammatory stress, shown a 1.46-fold increase; PD-L1, involved in peripheral tolerance via PD-1 engagement on T cells, increased by 1.38- fold. Whereas heat shock protein 70 (HSP-70), known to modulate both innate and adaptive immune responses, was upregulated in both TFF and _cyt_TFF_lyo_ -sEVs by 2.08- and 3.13-fold, respectively, compared to CM. (Fig. 1d) [4]. These findings underscore the potential of EVs to reprogram immune responses in autoimmune diseases, including AITD, by modulating interconnected inflammatory and tolerogenic pathways [5]. Finally, the protein–protein interaction network generated using STRING (https://string-db.org/) highlights a functional interplay between pro-inflammatory mediators and immunoregulatory proteins modulated by Th1-primed sEVs derived from f-LSCs in the activated PBMCs potentially assimilable with a context of autoimmune thyroid disease. The network reveals significant interconnections among key inflammatory factors (IL-2, IL-17, MCP-1, hnRNPA2/B1) and regulatory molecules (IL-4, IDO1, PD-L1, COX-2, HSP-70), suggesting that EV cargo can simultaneously suppress inflammatory pathways and promote immune tolerance (online resource).

In conclusion, this study demonstrates the immunomodulatory potential of sEVs derived from Th1-primed f-LSCs, capable of suppressing effector T cell proliferation and promoting Treg expansion in vitro Although a technical comparison of isolation methods was not the primary objective of this study, our findings support the superior performance of TFF over chemical precipitation in preserving the biological activity of EVs. This advantage may be attributed to the gentle, membrane-based separation mechanism of TFF, which minimizes vesicle aggregation and protein contamination, unlike polymer-based precipitation method. The preservation of bioactivity after lyophilization and the use of a multimodal characterization approach support the feasibility of sEVs as a cell-free therapeutic tool for early-stage Hashimoto’s thyroiditis. Further in vivo studies are needed to confirm their long-term immunoregulatory effects.

## Supplementary Information

Supplementary Material 1.

## Data Availability

All data supporting the findings of this study are available within the manuscript, supplementary matherial and on 10.6084/m9.figshare.30689240.v1.
