# Supplementary material for: Th-1 cytokine-primed small extracellular vesicles from limbal mesenchymal stem cells modulate immune and inflammatory responses in Hashimoto's Thyroiditis: an ex vivo proof-of-concept study
Source: Mol Biomed. 2025 Dec 15;6:139. doi: 10.1186/s43556-025-00388-y (PMC12705507; doi:10.1186/s43556-025-00388-y)
Supplement: Supplementary file 1 — Supplementary Material 1. [file 43556_2025_388_MOESM1_ESM.pdf]

Title Page

Article type: Letter

Title: Th-1 Cytokine-Primed Small Extracellular Vesicles from Limbal Mesenchymal Stem Cells Modulate Immune and Inflammatory Responses in Hashimoto's Thyroiditis: An Ex Vivo Proof-of-Concept Study

Authors: Laura Tomasello[1], Valentina Guarnotta[1,2,*], Giuseppe Siragusa[2,3], Mattia Biondo[3], Giorgio Arnaldi[1,2], Carla Giordano[1], Giuseppe Pizzolanti[1,2,4]

* Corresponding Author: Valentina Guarnotta; e-mail: valentina.guarnotta@unipa.it; telephone number: +390916552138

Author information

Laura Tomasello[1], PhD 1 Department of Health Promotion, Mother and Childcare, Internal Medicine and Medical Specialties, Laboratory of Endocrinology and Regenerative Medicine "Aldo Galluzzo", University of Palermo, Piazza delle Cliniche 2, 90127 Palermo, Italy

Valentina Guarnotta[1,2,*], PhD 1 Department of Health Promotion, Mother and Childcare, Internal Medicine and Medical Specialties, Laboratory of Endocrinology and Regenerative Medicine "Aldo Galluzzo", University of Palermo, Piazza delle Cliniche 2, 90127 Palermo, Italy; 2 University Hospital "Paolo Giaccone", Piazza delle Cliniche 2, 90127 Palermo, Italy.

Giuseppe Siragusa[2,3], MScs 2 University Hospital "Paolo Giaccone", Piazza delle Cliniche 2, 90127 Palermo, Italy; 3 Scienze e Tecnologie Biologiche Chimiche e Farmaceutiche (STEBICEF), University of Palermo, Viale delle Scienze, Ed. 16, 90128, Palermo, Italy.

Mattia Biondo[3], MD 3 Scienze e Tecnologie Biologiche Chimiche e Farmaceutiche (STEBICEF), University of Palermo, Viale delle Scienze, Ed. 16, 90128, Palermo, Italy.

Giorgio Arnaldi[1,2], MD 1 Department of Health Promotion, Mother and Childcare, Internal Medicine and Medical Specialties, Laboratory of Endocrinology and Regenerative Medicine "Aldo Galluzzo", University of Palermo, Piazza delle Cliniche 2, 90127 Palermo, Italy; 2 University Hospital "Paolo Giaccone", Piazza delle Cliniche 2, 90127 Palermo, Italy.

Carla Giordano[1], MD 1 Department of Health Promotion, Mother and Childcare, Internal Medicine and Medical Specialties, Laboratory of Endocrinology and Regenerative Medicine "Aldo Galluzzo", University of Palermo, Piazza delle Cliniche 2, 90127 Palermo, Italy.

Giuseppe Pizzolanti[1,2,4], PhD 1 Department of Health Promotion, Mother and Childcare, Internal Medicine and Medical Specialties, Laboratory of Endocrinology and Regenerative Medicine "Aldo Galluzzo", University of Palermo, Piazza delle Cliniche 2, 90127 Palermo, Italy; 2 University Hospital "Paolo Giaccone", Piazza delle Cliniche 2, 90127 Palermo, Italy. 4 ATeN Centre-Advanced Technologies Network Centre, Viale delle Scienze, Ed. 18, 90128, Palermo, Italy

* Corresponding Author: Valentina Guarnotta; e-mail: valentina.guarnotta@unipa.it; telephone number: +390916552138

Materials and Methods.

Collection and pre-processing tissue culture

Isolation of fibroblast-like Limbal Stem Cells (f-LSCs): The study was approved by the Ethical Committee of the AOUP, University of Palermo (No. 09/2009). Human corneo-scleral rings from donors were processed as previously described. Briefly, the bioptic samples underwent firstly mechanical dissection and were

subsequently enzymatically digested with collagenase I (5 mg/mL; Sigma-Aldrich, Merck KGaA, Darmstadt, Germany) overnight at 37°C in a shaking bath. The day after, the digest was centrifugated and the pellet placed in p60 culture dishes (Corning, New York, USA) in complete culture medium (DMEM/F12 supplemented with 5% embryonic stem cell-tested foetal bovine serum (EC-FBS; PAA Laboratories, Austria), 1x ITS (5g/mL insulin, 5g/mL transferrin, 5g/mL selenium; PAA Laboratories, Austria) and 20 ng/mL basic- fibroblast growth factor (b-FGF; Preprotech, UK) to start the cell culture. The f-LSCs cultures were kept in incubator at 37°C and 5% pCO_2. The medium was replaced every three days. F-LSCs until culture passage (p) 4 were used for all analysis.

General cell characterization

Spheroid forming assay: The f-LSCs (p3) were placed in ultralow-attachment six-well plates (Corning, Life Science, Italy) at a density of 1.5×10^3 cells/mL and kept in culture medium without serum up to 15 days at 37°C and 5% pCO_2. Spheroid establishment was assessed by the detection of spheric formation (limbo-spheres, number of cells > 3) with defined margin under a Zeiss Axiovert 135 optical microscope.

Immunofluorescent staining: Limbo-spheres were transferred into a cell-culture chamber slide (Labtek II; Nunc, Waltham, MA, USA) under adhesion-condition culture and incubated in complete culture medium for 2 hours, 6 and 24 hours, at 37 °C in 5% pCO_2. After incubation period, the limbo-spheres were fixed for 30 minutes in 2% (wt/vol) paraformaldehyde (PFA)/PBS at room temperature and incubated with 1 μg/ml anti-human SSEA-4 monoclonal antibody PE-conjugated (Miltenyi Biotec, Bergisch Gladbach, Germany). The samples were counterstained with 10μl 4′,6-diamidino-2-phenylindole (DAPI, Sigma Aldrich, Merck KGaA,Germany) to detect the nuclei and observed under a Leica DMI3000 B fluorescence microscope.

Th1 cytokine priming: Once reached the confluence, f-LSCs were kept in serum-free DMEM containing 1% sodium pyruvate, 1% L-glutamine and 20 ng/mL b-FGF. After 24 hours, f-LSCs were primed by supplementation with 20ng/mL IFN-γ and 10ng/mL IL-1β, up to 48 hours. All cytokines were purchased by PrepoTech, (Thermo Fisher Scientific, IT)

sEV isolation and lyophilization

Medium collection: 40 mL condition culture medium (CM) were collected from Th1-primed and no primed f-LSCs. CM were centrifugated at 500g for 10 min and firstly filtered through a 0.44μm cell strainer and subsequently trough a 0.22μm filter.

sEV isolation by tangential flow filtration (TFF): sEVs were isolated from 20mL of CM by TFF based device (HansaBioMed Life Sciences OÜ, Estonia) according to manufacturing information. Briefly, the CM was passed through a filter system of polyethersulfoine fibers with a pore size of 50±10nm and concentrated up to ten times. The 2mL sEVs concentrated suspension isolated by TFF (sEV_{TFF}) was stored at -20°C until use.

sEV isolation by chemical precipitation (CP): sEVs were isolated from 2mL of CM by Exo-prep kit, according to manufacturing information (HansaBioMed Life Sciences OÜ, Estonia). Briefly, CM was centrifugated at 300g for 10min, at 1200g for 20min and at 1000g for 30min and the supernatant was incubated with EXO-Prep solution. After 1 hour in ice, the sample was centrifugated at 10000g for 20 min, at 1500g for 2 min and finally sEVs isolated by CP (sEV_{CP}) was resuspend in 200µL PBS and stored at -20°C until use.

Single step freeze-drying process: sEV_{TFF} and sEV_{CP} were transferred to the Synthesis Laboratory, directed by Professor Licciardi, at the Aten Center of the University of Palermo and frozen at -80°C overnight. The day after lyophilization process was performed. Briefly, the tubes were opened and covered with a film in which 6 holes were pierced (1 mm diameter each), and loaded into a lyophilizer (Labconco, Freeze Zone, Labconco Corporation, MO, USA) within a freeze-dryer shelf precooled at −50°C and 0.35 mbar vacuum pressure. In lyophilization session, we used 2 mL of sEV_{TFF} and 200µL of sEV_{CP} suspension. The lyophilization process was stopped after 16 and 10 hours for sEV_{TFF} and sEV_{CP} suspension, respectively. The lyophilized sEVs were rehydrated with DMEM7F12 without serum and used for downstream analysis.

sEV characterization

Size distribution and quantification of sEVs: sEV_{CP} and sEV_{TFF} suspensions were diluted 1:100 in PBS and acquired by Zetasizer nano ZSP 2 instrument (Malvern, Malvern,UK). Size distribution and quantification were detected by dynamic light scattering (DLS) performed on Zetasizer v.7.11 software (Malvern, Malvern,UK). The measurement parameters set was measurement type “size”, material refractive index (RI) “1.59”, absorption “0.01”, material “polystyrene latex”, dispersant “water”, dispersant RI “1.33”, temperature “25°C, viscosity “0.88 cP, duration “60 s”, measurement position “3”, cell description disposable micro cuvette “40 µl”, data processing size “distribution by intensity”.

sEV marker characterization by flow cytometry: The staining for sEV markers, CD63 and CD8, was performed by Exo-FACS following the manufacturer’s instructions (HansaBioMed Life Sciences OÜ, Estonia). All reaction mixtures were then acquired using a FACS Calibur flow cytometer (Becton-Dickinson, New Jersey, USA) and analysed with the CellQuest Pro software.

Western blot analysis: Protein from sEV_{TFF} and sEV_{CP} were extracted using RIPA lysis buffer and quantified by Qubit™ Protein Assay according to manufacture instruction (catalog number Q33211, ThermoFisher Scientific, Milano, Italy) on Qubit fluorometer instrument. Non-reducing Laemmli buffer was added to protein samples and heated for 5 min at 95 °C; 20 µg of proteins were loaded on MiniProtean TGX Precast gels (Bio-Rad Laboratories, Inc., Italy) at 200 V for 35 min; the protein transfer to western blot membrane was performed using a Bio-Rad's Trans-Blot® Turbo™ System within 3 min transfer protocol. Trans proteins were blotted with anti-human primary unconjugated antibodies of interest (Table 1) and incubated overnight. The day after western blot membranes were blocked and incubated with secondary antibody (goat anti-mouse IgG-HRP, Bio-Rad Laboratories, Inc., Italy) at room temperature for two hours. The protein-antibody complexes were revealed by the ECL prime (Amersham, Milan, Italy) on a CCD camera (Chemidoc, Bio-Rad, Italy). Western

blot bands were quantified with ImageJ version 1.48 software (National Institutes of Health, Bethesda, MD, USA).

Table1 Antibodies list used to perform western blot assay

ANTIBODY	Brand/code	DILUTION
Anti-human-CD8	HansaBiomed Life sciences /HBM-CD81-EM4-100	10 µg/mL
Anti-human-CD	HansaBiomed Life sciences /HBM-CD9-EM4-100	10 µg/mL
Anti-human-Ali	HansaBiomed Life sciences /HBM-Alix-EM4-100	10 µg/mL
Anti-human-CD63	HansaBiomed Life sciences /HBM-CD63-EM4-100	20 µg/mL
Anti-human-TXN-1	Novus Biologicals /AF1970	0.1 µg/mL
Anti-human-COX-2	Novus Biologicals /NB100-689	1:100
Anti-human-PD-L1	Novus Biologicals/AF156	2 µg/mL
Anti-human-HSP-70	Novus Biologicals /NBP1-97490	1:5000
Anti-human-Actin-β	Novus Biologicals /NB600-501	1:10000
HRP-tagged secondary ab (mouse)	Santa Cruz Biotechnologies	1:10 000

Functional studies

Selection Patient and peripheral blood mononuclear cells (PBMCs) collection

Five female patients aged 28–44 with isolated HT and 5 female healthy controls (HC) aged 24–32, were included. All patients provided informed consent, in accordance with the Declaration of Helsinki. The study was approved as No. 07/2018, as previously described. Both patients and controls had a condition of euthyroidism (mean TSH 2.35±0.63 microU/mL vs. 2.30 ±0.55 microU/mL, respectively). However, all patients with HT were on replacement thyroid treatment, at the mean dose of 1.3 microg/kg/day. PBMCs of HT and five patients with HT were isolated from venous blood by conventional Ficoll.

Proliferation assay and cell cycle analysis.

Activated PBMCs were treated with 20 or 80µg/mL of lyophilized sEVs, derived from Th-1 primed f-LSCs ($_{cyt}EV_{lyo}$) and incubated up to 72 hours. The percentage of proliferation was determined by ki67/$^{+}$bcl2^{+} cells

by cytofluorimetric assay. Intracellular staining for bcl2 and and Ki67 was performed using BD Cytofix/Cytoperm™ Plus Fixation/Permeabilization Kit (BD Biosciences, Italy) according to the manufacturer's instructions. After incubation with the appropriate secondary antibodies (Alexa Flour 488- or PE-conjugated anti mouse IgG, Thermo Fisher Scientific, Italy) for 1h at 4°C, the samples were acquired using FACS Calibur and data analysed by CELL Quest Pro software (BD Pharmingen, San Jose, CA).

T cell phenotyping

1×10^6 cells/mL single cell suspension of activated HC and HT-PBMCs, with or without ${}_{cyt}EV_{lyo}$, was used for each marker detection. The cells were incubated with FcR blocking reagent (Miltenyi Biotec, Bergisch Gladbach, Germany) and then incubated with primary antibody at 4°C for 30min in the dark. After incubation, PBMCs were fixed with 2% paraformaldehyde (PFA) and washed with staining buffer (PBS calcium and magnesium free, Euroclone S.p.A., Italy), supplemented with 1% BSA, Sigma-Aldrich, Merck KGaA, Germany). For detection of intracellular marker BD Cytofix/Cytoperm Plus Fixation/Permeabilization Kit (BD Biosciences, Milan, Italy) was used according to the manufacturer's instructions. For the gating strategy, lymphocyte populations were gated based on light scattering characteristics using side scatter (SSC) and forward scatter (FSC). All data were acquired on a FACSAria™ III Cell Sorter and analyzed using FACSDiva™ 7 software (BD Pharmingen, San Jose, CA Italy). The antibodies used for phenotyping were listed in table 2.

Table 2 Primary antibodies list used for cytofluorometric analysis.

**Primary antibody**	**Product**	**Code**
CD4	Clone SK3 BD™ CD4 FITC	664528, BD Horizon Biosciences
Foxp3	Clone 259D/C7 PE-CF594	562421, BD Horizon Biosciences
CD25	Clone M-A251 PerCP-Cy™5.5	561398, BD Horizon Biosciences

Gene expression analysis

Total RNA from PBMCs was extracted using the RNeasy Micro Kit (Qiagen, Italy), according to the manufacturer's protocol. Quantitative and qualitative analysis was carried out on Nano Drop 2000 spectrophotometer (Thermo Scientific, Italy). Two µg of total RNA was reverse transcribed with Oligo dT primers (Promega Italia s.r.l., Italy) and ImProm-II™ Reverse Transcription System (Promega Italia s.r.l., Italy). The primer sequences and details are listed in Table S3. All reactions were performed using the Quantitect SYBR Green PCR Kit (Qiagen, California, USA) on the RotorGS4ene Q Instrument (Qiagen, California, USA). Each reaction was performed at least in triplicate. The specificity of the amplified products was determined by the melting peak analysis. Relative mRNA expression for each gene was analysed using the ΔΔCt method. The results are represented on GraphPad Software, Inc, California.

Table 3 The primer sequence list used for mRNA expression analysis.

Gene	Product Name/QuantiTect Primer	GeneGlobe Id
**IFN-γ**	Hs_IFNG-1_1	QT00000525
**IL-4**	Hs_IL4_1	QT00012565
**IL-17A**	Hs_IL17A_1	QT00009233
**IDO**	Hs_IDO1_1	QT00000504
**FAS-L**	Hs_FASLG_1	QT00001281
**PD-L1**	Hs_CD274_1	QT00082775
**hnRNPA2/B1**	Hs_HNRNPA2B1_1	QT00070931
**MCP-1**	Hs_CCL2_1	QT0021273

Statistical Analysis

Data were expressed as mean ± SD. Statistical comparison between groups was conducted according to one-way ANOVA with Tukey multiple comparison test using GraphPad Prism 5 software (San Diego, CA, USA). Statistically significant differences were defined at $p< 0.05$ with a 95% confidence interval (statistical significance were indicated as follows: *p <0.01, **p < 0.05, ***p < 0.001).

Artwork and Illustration

Photoshop CS4 was used to create all figures.
